# Overexpression of X-Box Binding Protein 1 (XBP1) Correlates to Poor Prognosis and Up-Regulation of PI3K/mTOR in Human Osteosarcoma

**DOI:** 10.3390/ijms161226123

**Published:** 2015-12-02

**Authors:** Jielai Yang, Dongdong Cheng, Shumin Zhou, Bin Zhu, Tu Hu, Qingcheng Yang

**Affiliations:** 1 Department of Orthopedics, Shanghai Jiao Tong University Affiliated Sixth People’s Hospital, No. 600, Yishan Road, Shanghai 200233, China; jielai1990@163.com (J.Y.); 18817314717@163.com (D.C.); zhubin.0526@163.com (B.Z.); zzuhutu@163.com (T.H.); 2 Institute of Orthopaedics, Shanghai Jiao Tong University Affiliated Sixth People’s Hospital, No. 600, Yishan Road, Shanghai 200233, China; zhoushumin168@hotmail.com

**Keywords:** X-box binding protein 1, growth, survival, PI3K/mTOR signaling, osteosarcoma

## Abstract

Increasing evidence demonstrates that dysregulation of XBP1 function contributes to tumorigenesis in some cancers. However, little is known about the role of XBP1 in the progression of osteosarcoma (OS). The expression of XBP1 in OS samples was measured by quantitative RT-PCR and Western blotting assays. Cell cycle analysis and cell counting kit 8 (CCK8) assays were performed to determine the effects of XBP1 expression on cells growth capacity. Cell apoptosis coassay was applied to determine cell survival. The expression of genes affected by XBP1 was examined by quantitative RT-RCR and validated by Western blotting assays. XBP1 was overexpressed in OS clinical samples compared with corresponding non-cancerous tissues. Overexpression of XBP1 was significantly associated with advanced clinical stages, high degree of malignancy and low tumor necrosis rate. Furthermore, hypoxia activated XBP1, and silencing XBP1 significantly enhanced OS cell apoptosis. Knock-down of XBP1 resulted in inhibition of OS growth. Most importantly, knockdown of XBP1 led to down-regulation of PIK3R3 and mTOR. Taken together, XBP1 is up-regulated and has a pro-tumor effect in OS with activation of PI3K/mTOR signaling. Thus, targeting XBP1 may provide a new potential therapeutic method for OS.

## 1. Introduction

Osteosarcoma (OS) is the most frequent primary bone malignant neoplasm in children and young adults, which is of high propensity of local invasion and distant metastases [1,2]. Despite current advances in treatments, comprising chemotherapy and wide excision of tumors, the recurrence rate of patients with localized or metastasis disease at diagnosis is high [3,4]. Though the survival rate has increased approximately fourfold from 1960s to 1980s, it remains almost unchanged during the recent three decades [5]. Thus, new treatment is eagerly needed.

Tumor cells can induce a series of adaptive response signaling pathways to survival in the condition of deprivation of oxygen, ATP, or other essential nutrients [6]. One such adaptive pathway is the endoplasmic reticulum (ER) stress response. Inositol-requiring enzyme-1α (IRE1α)/X-box binding protein 1 (XBP1) axis, a vital branch of ER stress response, exists in both invertebrate and vertebrate cells [7,8]. Human X-box binding protein 1 exists in two different forms, spliced (XBP1s) and un-spliced (XBP1u) isoforms [9], which is involved in a variety of human physiological and pathologic processes such as lipogenesis [10,11], adipogenesis [12], atherosclerosis [13], and ischemia [14]. 

Previous studies reported that XBP1 was activated in various human cancers, including both mesenchymal and epithelial cancers [15,16,17,18]. In multiple myeloma (MM), XBP1s overexpression in bone marrow stromal cells is critical for myeloma cell growth and osteoclast formation, and the increased ratio of XBP1s to XBP1u predicts poor outcome of myeloma patients [19,20]. In breast cancer, XBP1 was activated and correlated with poor prognosis in triple-negative breast cancer patients [18]. As for the biological mechanisms, the hypoxia-inducible factor 1 (HIF-1)/Vascular endothelial growth factor A (VEGF-A) signaling was most investigated. However, recent researches in human endothelial cells suggest a new mechanism that XBP1 is involved. Martin *et al.* observed that XBP1 protected endothelial cells from oxidative stress through interaction with histone deacetylase 3, which form a complex with Akt1 and mTOR [21]. Zeng *et al.* indicated that VEGF-induced XBP1s regulated endothelial cell growth in a PI3K/Akt/GSK3β/β-catenin/E2F2–dependent manner [22]. Though these findings were not reported in tumor cells, it provided us new perspectives to investigate cancers. 

The role of XBP1 in OS progression is unknown. In this study, we found that the overexpression of XBP1 in human OS. Moreover, we find that the expression levels of XBP1 correlated with clinical stages in a cohort of OS patients. We also discovered that knockdown of XBP1 resulted in growth inhibition but promoted apoptosis of OS cell lines. Most importantly, we found that PI3K/mTOR signaling was involved in the process of XBP1-regulated OS progression, which suggests a novel mechanism of XBP1’s role in OS. Therefore, XBP1 may be a novel target for OS treatment.

## 2. Result

### 2.1. XBP1 Expression Was Up-Regulated in OS Clinical Samples and Associated with the Progress of OS

**Figure 1:**
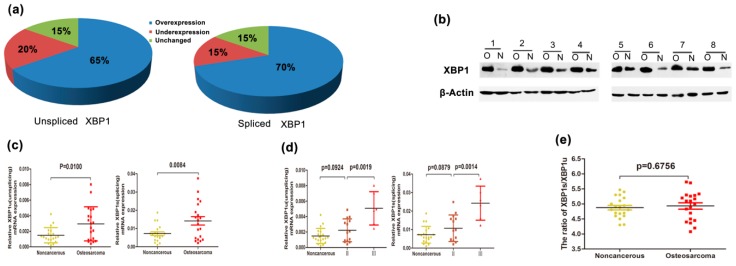
XBP1 is up-regulated in OS and correlated with the advanced clinical stage. (**a**) Relative expression of XBP1u and XBP1s were detected by RT-PCR in 20 pairs of OS clinical samples and their matched noncancerous tissues. The up-regulated XBP1u and XBP1s were, respectively, observed in 65% and 70% OS samples compared with the corresponding non-cancerous tissues; (**b**) XBP1 was overexpressed in OS clinical samples; Western blot tests showed XBP1 protein was higher in eight OS samples than their corresponding non-cancerous tissues. O: osteosarcoma; N: noncancerous; (**c**) Up-regulation of XBP1u and XBP1s was observed in 20 pairs OS comparing to their corresponding noncancerous tissue; (**d**) XBP1u and XBP1s mRNA expression in different clinical stages of OS; (**e**) The ratio of XBP1u to XBP1s. The dots in different color and sharp were used to differentiate different clinical stage. (Yellow for noncancerous tissue, Gray for stage II, Red for stage III). The patients were staged in accordance with the Ennecking musculoskeletal tumor staging system. Error bars represent mean ± s.d. of triplicate experiments. The statistical analysis was performed using paired *t*-test (**c**,**e**) and Student’s *t*-test (**d**).

Previous studies showed that XBP1 was overexpressed and correlated with clinical progress in multiple cancers, including the myeloma and breast cancer [18,19]. To investigate whether XBP1 was overexpressed and involved in the progression of OS, we detected the mRNA expression of XBP1 (both un-spliced and spliced) in 20 pairs of human OS and their corresponding normal tissues. The correlation between XBP1 expression and the data of OS patients was shown in Table 1. The XBP1 mRNA expression was not correlated to age, gender, anatomic location, or tumor size statistically significant. However, there was a significant correlation of XBP1 expression with clinical stage (*p* < 0.01), degree of malignancy (*p* < 0.05), and tumor necrosis rate (*p* < 0.05). In addition, XBP1u and XBP1s were overexpressed, respectively, in 65% and 70% of OS tissues (Figure 1a), both isoforms unregulated almost twofold (Figure 1c) in OS compared with non-cancerous tissues. However, the ratios of XBP1s to XBP1u of the two groups were similar (Figure 1e). We also observed a significant increase of XBP1 mRNA in advanced clinical stages compared with early clinical stage (Figure 1d). More importantly, we extracted proteins from eight fresh OS specimens and their corresponding non-cancerous tissues, and observed that XBP1 protein was up-regulated in all of the eight OS tissues compared with their corresponding non-cancerous tissues (Figure 1b). Taken together, these results indicate that XBP1 is up-regulated and potentially had a pivotal role in the growth and survival of OS.

**Table 1:** Relationship between XBP1 mRNA expression and their clinicopathologic parameters in 20 of osteosarcoma.

Parameters	*N* (%)	Median Expression of XBP1u		Median Expression of XBP1s	
		Mean ± s.d.	*p*-Value	Mean ± s.d.	*p*-Value
Age (years)					
<20	12 (60%)	0.0027 ± 0.0019	0.5053	0.0126 ± 0.0085	0.4340
≥20	8 (40%)	0.0034 ± 0.0026		0.0164 ± 0.0127	
Gender					
Male	14 (70%)	0.0032 ± 0.0020	0.1031	0.0166 ± 0.0100	0.0993
Female	6 (30%)	0.0016 ± 0.0016		0.0084 ± 0.0091	
Anatomic location					
Tibia/Femur	14 (70%)	0.0027 ± 0.0021	0.5421	0.0132 ± 0.0099	0.5277
Elsewhere	6 (30%)	0.0034 ± 0.0025		0.0164 ± 0.0114	
Clinical stage					
II	14 (70%)	0.0020 ± 0.0015	0.0019 **	0.0098 ± 0.0073	0.0014 **
III	6 (30%)	0.0051 ± 0.0022		0.0243 ± 0.0092	
Tumor size (cm^3^)					
<50	10 (50%)	0.0033 ± 0.0025	0.4580	0.0117 ± 0.0086	0.3094
≥50	10 (50%)	0.0025 ± 0.0018		0.0165 ± 0.0115	
Degree of malignancy					
Low	9 (45%)	0.0018 ± 0.0015	0.0109 *	0.0091 ± 0.0076	0.0099 **
High	11 (55%)	0.0042 ± 0.0022		0.0203 ± 0.0098	
Tumor necrosis rate (%)					
<90	11 (55%)	0.0035 ± 0.0025	0.0450 *	0.0192 ± 0.0103	0.0103 *
≥90	9 (45%)	0.0015 ± 0.0012		0.0079 ± 0.0060	

Clinical stage is classified by Ennecking-Musculoskeletal Tumor Staging System. *p*-value represents the probability from a Student’s *t*-test for XBP1 mRNA expression between variable subgroups. * *p* < 0.05, ** *p* < 0.01, which was considered to have a significant difference.

### 2.2. Knockdown of XBP1-Inhibited OS Cell Proliferation Following Hypoxic Treatment

**Figure 2:**
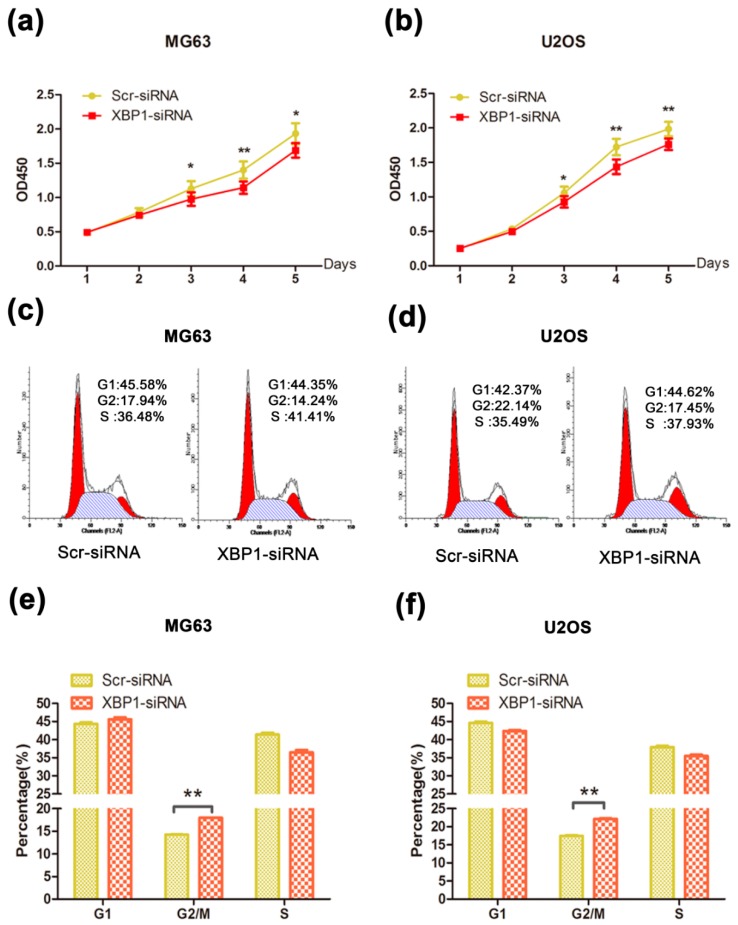
(**a**,**b**) Knockdown of XBP1 inhibited the growth and proliferation in OS cells. The viability of MG63 and U2OS was examined by CCK8 assay at different time points as indicated; (**c**,**d**) Flow cytometry of cell distribution after transfection; (**e**,**f**) Histograms of each phase in cell cycle of MG63 and U2OS. Silencing XBP1 increased cell proportion of G2/M phrase in MG63 and U2OS cells. Data represent mean ± s.d. Each performed in triplicate. * *p* < 0.05, ** *p* < 0.01, by Student’s *t*-test. Transfected cells was treated in the hypoxia (24 h) chamber before the assays.

To explore the potential role of XBP1 in OS tumorigenesis, we knocked down XBP1 by siRNA that targets both isoforms of XBP1. We performed CCK-8 assays, and observed that after knockdown of XBP1, the viabilities of MG63 and U2OS cells decreased (Figure 2a,b). In addition, knockdown of XBP1 delayed the cell cycle progression, both MG63 (Figure 2c,e) and U2OS cell (Figure 2d,f) showing G2/M phase arrest. To summarize, these results indicate that XBP1 is required for OS cell growth.

### 2.3. Silencing XBP1-Attenuated OS Cells’ Survival under Both Normoxic and Hypoxic Condition

**Figure 3:**
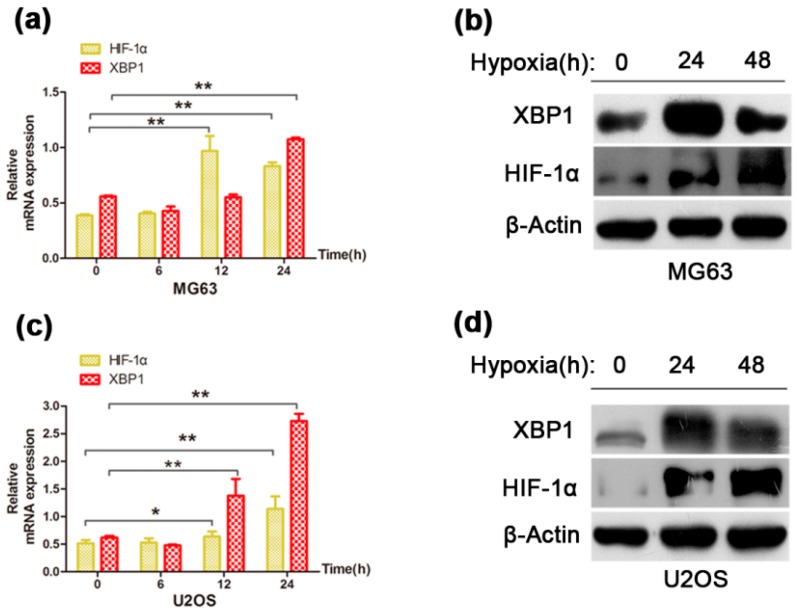
The up-regulation of XBP1 under hypoxia condition in OS cells. (**a**,**c**) The mRNA expression of XBP1 and HIF-1α in MG63 and U2OS cells for indicated h of hypoxia (1% O_2_) treatment; (**b**,**d**) The protein expression of XBP1 and HIF-1α in MG63 and U2OS cells for indicated h of hypoxia treatment. HIF-1α expression demonstrated specificity of treatment to hypoxia. β-Actin was used as an internal control. Error bars represent mean ± s.d. of triplicate experiments.* *p* < 0.05, ** *p* < 0.01 by Student’s *t*-test.

To identify whether XBP1 was activated in OS cells under hypoxic condition, we treated MG63 and U2OS cells with hypoxia (1% O_2_) condition. The XBP1 mRNA expression increased significantly in MG63 and U20S cells after 24 h hypoxia (Figure 3a,c). We next examined the protein levels of XBP1 after hypoxia (1% O_2_) treatment for 24 h and 48 h. In consistent with the mRNA levels, protein levels of XBP1 elevated significantly by hypoxia 24 h, but subsequently decreased in 48 h (Figure 3b,d), which indicated that the activation of XBP1 reach to a peak for about 24 h hypoxic treatment.

**Figure 4:**
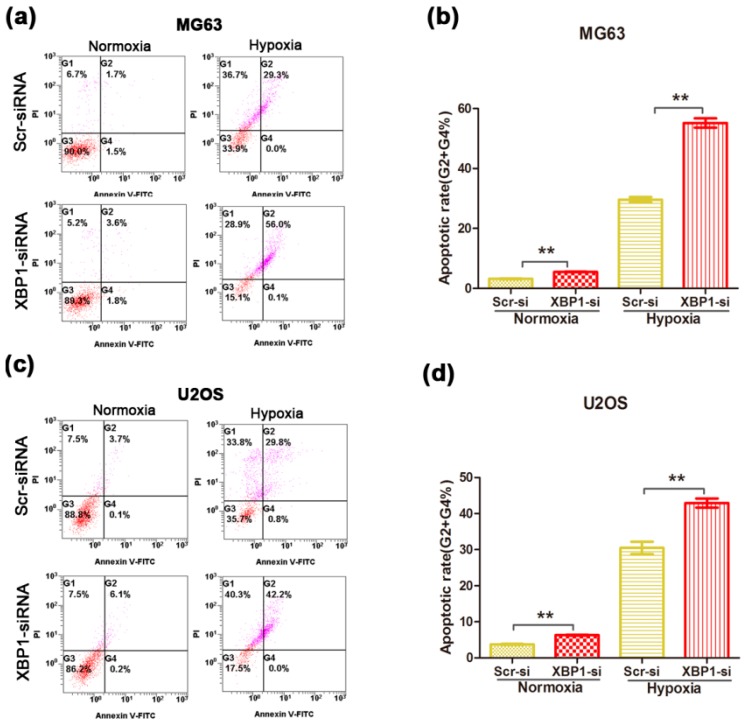
Knockdown of XBP1 increased the apoptosis rate of OS cells under both normoxic and hypoxic condition. (**a**,**b**) Comparison of apoptosis between Scramble and XBP1-knockdown group in MG63 under normoxic and hypoxic condition; (**c**,**d**) Comparison of apoptosis between Scramble and XBP1-knockdown group in U2OS under normoxic and hypoxic condition. The data represent mean ± s.d. of triplicate (**b**,**d**). ** *p* < 0.01 by Student’s *t*-test. The cells were transfected with XBP1 siRNA (50 nM) for 48 h, and treated with or without hypoxia (1% O_2_) for 24 h. All the apoptotic rates were measured by using the fluorescein isothiocyanate Annexin V apoptosis assay. PI, propidium iodide.

Next, to determine the role of XBP1 in cell survival under normoxic and hypoxic conditions, we compared the apoptotic rates between the control group and XBP1-knockdown group. Under normoxic condition, silencing XBP1 promoted cell apoptosis in OS cells, but the effect was mild (Figure 4a,c). After 24 h of hypoxia, the apoptotic rate increased significantly in two OS cells and the pro-apoptosis effect was more obvious in the XBP1-knockdown group (Figure 4b,d). In general, these data suggest that XBP1 is activated under hypoxic conditions, and this activation is essential for OS cell survival in hypoxic conditions.

### 2.4. Knockdown of XBP1 Down-Regulated the Expression of PIK3R3/mTOR in OS Cells

**Figure 5:**
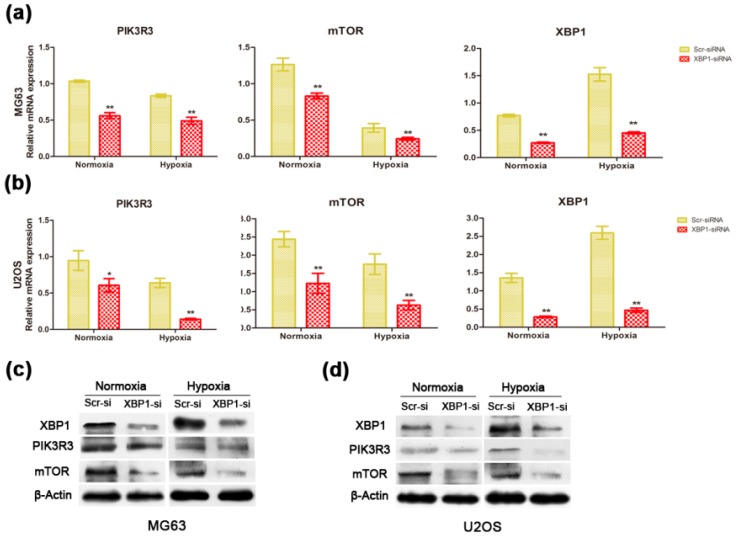
Down-regulation of PIK3R3 and mTOR after inhibiting the expression of XBP1**.** (**a**,**b**) The mRNA expression of PIK3R3, mTOR, and XBP1 between the scrambled and XBP1-knockdown group under both normoxic and hypoxic condition in MG63 and U2OS cells; (**c**,**d**) The protein expression of PIK3R3, mTOR, and XBP1 between the scrambled and XBP1-knockdown group under both normoxic and hypoxic condition in MG63 and U2OS cells. Transfection efficacy of XBP1-siRNA was confirmed by qRT-PCR and Western blot assays. The data are representative of three independent experiments. Error bars represent mean ± s.d. * *p* < 0.05, ** *p* < 0.01 by Student’s *t*-test.

To investigate a potential mechanism by which XBP1 affects the proliferation and apoptosis of OS cells, a series of pathways functioning in the stressful condition were detected. After some initial screening work, we found that knockdown of XBP1 decreased the mRNA levels of PIK3R3 and mTOR in MG63 (Figure 5a) and U2OS cells (Figure 5b) under both normoxic and hypoxic conditions. In addition, we detected the protein expression of PIK3R3 and mTOR after knockdown of XBP1. The result was consistent with those of mRNA expression (Figure 5c,d). Taken together, these data show that the expression of PIK3R3 and mTOR was affected by XBP1 in OS cells, which indicated that XBP1 might play a crucial role in the growth and apoptosis by influencing PI3K/mTOR signaling.

## 3. Discussion

Accumulating evidence has demonstrated that ER-stress plays an indispensable role for tumor cells growth and survival under various stressors [23,24]. The IRE1α-XBP1 pathway is one of the most important ER-stress response pathways that are implicated in tumor growth, metastatic progression, and chemo-resistance [25]. In previous studies, activation of XBP1 was reported and correlated with clinical outcome in breast cancer [18,26]. Tumor growth and survival was severely compromised by blocking the expression of XBP1 in human pancreatic adenocarcinomas under hypoxic condition [27]. Multiple studies had demonstrated that IRE1α-XBP1 pathway is implicated in the pathogenesis of MM and XBP1 is a promising therapeutic target in treating MM [28,29,30]. Furthermore, XBP1 was involved in the progression of chemotherapy resistance in MM [31], and a recent study got the opposite result [32], suggesting the complexity of its role in regulating this function.

In the present study, we showed that XBP1 was significantly elevated in OS clinical samples at both mRNA and protein levels. The mRNA level of XBP1 correlated with advanced clinical stages, high degree of malignancy, and low tumor necrosis rate in OS patients. However, the ratio of XBP1s to XBP1u was similar between OS tissues and their matched non-cancerous ones (Figure 1e), which was different from previous results from MM [19] and breast cancer [18]. There are several possible reasons explaining the result: firstly, XBP1u and XBP1s might function in a synchronous effect in OS, which indicating a new mechanism for ER activity. For most studies, XBP1s or XBP1u was solely investigated regarding the activation of the IRE1 pathway and the XBP1s/XBP1u ratio was not regarded as an essential index. Secondly, the sample size may be a factor resulting in the different result. Previous studies of MM and breast cancer analyzed the ratio in a huge population sample, thus, more comprehensive clinical studies were required to get a more convincing outcome in OS. Taken together, the clinical results suggested that XBP1 might be involved in the growth of OS.

XBP1 was known to be activated under stressful conditions in various cancer cells [33,34]. Our *in vitro* data showed that knockdown of XBP1 resulted in a moderate inhibition of cellular growth and a mild apoptotic effect in OS cells under normoxic condition. However, when exposed to hypoxic conditions, XBP1 knockdown significantly promoted the apoptotic rate, which was inconsistent with elevated expression of XBP1 for hypoxia treatment. Though the transfection efficacy of siRNA varied in two OS cells, 70% in MG63 cells and 80% in U2OS cells, the effect of XBP1 on the growth and survival of OS was similar and was enhanced by hypoxia condition. These findings suggest that XBP1 is required for OS growth and survival under hypoxia, which was similar with previous studies in fibrosarcoma [16]. Further *in vivo* study is needed to validate the role of XBP1 in OS on a mouse model.

Though the importance of XBP1 has been indicated by studies with various cancer cells, the detailed mechanism underlying the importance remains largely unknown. The most widely accepted target of XBP1 was CHOP, which plays a crucial role in the apoptosis pathway [35]. For the study of MM, knockdown of XBP1 greatly compromised the expression of VCAM-1, IL-6, and RANKL in BM stromal cells [17], which were key players in supporting MM cell growth and destroying the bone. For breast cancer, XBP1 promoted triple-negative breast cancer by regulating HIF-1α pathway [18]. For human glioma, inhibition of IRE1α resulted in down-regulating of VEGF-A, IL-1β, IL-6, and IL-8 [36]. Other cancers, like liver and colon cancers [37,38], were also reported to be with activation of XBP1, however, the specific mechanisms were seldom explored.

Increasing evidence indicates that XBP1 has critical roles in the tumorigenesis and progression of various cancer cells [39,40]. Romero-Ramirez *et al.* observed that XBP1 was crucial for tumor cell survival under severe hypoxic condition, but it was activated in a HIF-1α-independent manner [16]. To understand the mechanism underlying the promoting effect of XBP1 in OS, we focused on pathways that are activated by stressful conditions. Thus, the HIF-1 signaling pathway, which has been reported to be activated in fibrosarcoma [16] and breast cancer [18], and has been proposed to be a general stress responsive pathway [41,42], was investigated. However, knockdown of XBP1 result in slightly down-regulation of HIF targets, like VEGF-A (Figure S1). We then considered PI3K/mTOR signaling pathways, which played an important role in cell growth and survival [43,44]. Furthermore, recent studies in endothelial cells showed that XBP1s may regulate cell proliferation and growth in a PI3K/Akt/GSK3β/β-catenin/E2F2-dependent manner [22]. Martin *et al.* [21] showed that XBP1, interacting with HDAC3, exert a protective effect on oxidative stress by up-regulating mTORC2-dependent Akt1 phosphorylation and Nrf2-mediated HO-1 expression. In this report, we observed that the expression of PIK3R3 and mTOR was down-regulated when inhibiting XBP1 expression, which was consistent with previous studies in endothelial cells [21,22]. Our results suggest that XBP1 exerts its effect on OS probably by influencing PI3K/mTOR signaling, which could be a novel mechanism by which XBP1 promotes cancer progression. However, the exact mechanism that XBP1 is required for the activation of PI3K/mTOR blurs, and further investigation is warranted.

## 4. Materials and Methods

### 4.1. Cell Culture and Hypoxia Treatment

Two OS cell lines (U2OS and MG63) were maintained at 37 °C in a humidified air atmosphere containing 5% CO_2_ in RPMI-1640 and DMEM media respectively. 10% fetal bovine serum (Biowest, Kansas, MO, USA), 100 U/mL penicillin, and 100 mg/mL streptomycin (Sigma-Aldrich, St. Louis, MO, USA) were added in the media. For hypoxia experiments, cells were treated at 60% to 70% confluence and maintained in a hypoxic chamber with 1% O_2_.

### 4.2. Human OS Samples

From 2014 to 2015, the 20 paired human OS clinical samples, including 20 OS samples and 20 non-cancerous tissues, were collected at the time of surgery at the department of bone oncology. Once the tumor was resected, specimens were selected, and put into tiny tubes. The tubes were then frozen in liquid nitrogen and stored at −80 °C in the refrigerator for a long period. All procedures involving human specimens were performed with written informed consent according to the Declaration of Helsinki and the research was approved by the Ethics Committee of the Sixth Peoples’ Hospital of Shanghai Jiao Tong University.

### 4.3. RNA Isolation and qRT-PCR Assays

Total RNA of human tissue samples and cultured cells was extracted and quantified with Trizol Kit (Invitrogen, Carlsbad, CA, USA) and Nanodrop 2000 (Thermo Fisher Scientific, Waltham, MA, USA), respectively, according to the manufacturer’s protocol. The PrimeScript RT Reagent kit (TaKaRa, Shiga, Japan) was used to synthesize the first-strand cDNA. RT-PCR was performed with SYBR Green premix Ex Taq (TaKaRa). Sequences of used primers were detailed in Table S1. β-Actin was used as internal control. 

### 4.4. siRNA Transfection

The siRNAs were synthesized by BioMics. The cells were transfected with siRNA using Lipofectamine 2000 Reagent (invitrogen) following the manufacturer’s protocol. The sequence of siRNA targeting XBP1 was 5’-CAACCCUGAAUUCAUUGUCUdTdT-3’. Transfection with the final concentration of 50 nM siRNA was conducted when the cell density was approximately 40% in six-well plates. 

### 4.5. Cell Proliferation and Cell Cycle Analysis

Cell Counting Kit-8 (CCK-8) Dojindo Molecular Technologies, Inc, Kumamoto, Japan) was used to measure the cell viability. In short, transfected cells previously treated in the hypoxia (24 h) chamber were seeded at the density of 3000 or 2500 per well in 96-well microplates. 10 μL of CCK8 solution and 100 μL DMEM was added to each well and incubated for 2 h. The optical density was detected at a wavelength of 450 nm by microplate reader (Model 680, Bio-Rad Laboratories, Hercules, CA, USA). This procedure was repeated once a day for five days. For cell cycle assay, transfected cells in the hypoxia chamber (24 h) were fixed in 70% ethanol at −20 °C in the refrigerator for 12–24 h. After that, cells were treated with staining solution, which containing 50 μg/mL propidium iodide (PI) (Biolegend, San Diego, CA, USA) and 50 μg/mL RNase A (BD LSRII, San Jose, CA, USA). Each experiment was repeated three times.

### 4.6. Cell Apoptosis Analysis

For the apoptosis analysis, 48 h post transfection, hypoxia-treated cells were placed into a hypoxia chamber for 24 h, normoxic cells were plated at the same density and maintained in 37 °C incubator. The fluorescein isothiocyanate Annexin V Apoptosis Detection kit I (BD Pharmingen, San Diego, CA, USA) was used. Briefly, the cells were collected and centrifuged at 2000× *g* for 5 min. The cells were then suspended in 500 μL binding buffer, supplemented with 5 μL Annexin V and 5 μL propidium iodide (PI), for 15 min of dark treatment at the room temperature. The flow cytometry (FC500 MPL, Beckman Coulter, Brea, CA, USA) was used to analyze the samples.

### 4.7. Western Blotting Analysis

The procedures were performed as described previously [18]. Briefly, total protein of cells and fresh tissues were isolated and separated by 6%–8% SDS-PAGE gels. Following the standard procedure, the blocking proteins in the nitrocellulose filter membranes were incubated with primary antibodies against the following antigens: XBP1 (Santa Cruz Biotechnology, Dallas, TX, USA), HIF1α (Santa Cruz Biotechnology), PIK3R3 (Abgent, San Diego, CA, USA), mTOR (Abgent), β-Actin (Sigma-Aldrich, New York, NY, USA). The secondary antibody was horseradish peroxidase-conjugated anti-rabbit IgG (Sigma-Aldrich). SuperSignal West Femto Maximun Sensitivity Substrate (Thermo Fisher Scientific, Waltham, MA, USA) was used in the subsequent visualization.

### 4.8. Statistical Analysis

Data was imaged with GraphPad Prism 5 software (Graphpad Software, Inc., La Jolla, CA, USA). Quantitative variables were presented as means ± standard deviation. A two-tailed Student’s *t*-test was used to compare the differences between two groups. The SPSS version 16.0 (SPSS, Inc., Chicago, IL, USA) software was used to analyze the data. *p* < 0.05 was considered statistically significant different.

## 5. Conclusions

We demonstrated for the first time that XBP1 was overexpressed in human OS tissues. High levels of XBP1 correlated with advanced clinical stages, high degree of malignancy, and low tumor necrosis rate in OS. In culture OS cells, knockdown of XBP1 inhibited cell growth and survival. More importantly, XBP1 expression correlates to up-regulation of PI3K/mTOR, a signaling pathway critical for cell growth and proliferation. These data indicate that XBP1 may be a prognostic biomarker and therapeutic target for OS.

## Supplementary Materials

Supplementary materials can be found at http://www.mdpi.com/1422-0067/16/12/26123/s1.
