# Supplementary material for: Overexpression of X-Box Binding Protein 1 (XBP1) Correlates to Poor Prognosis and Up-Regulation of PI3K/mTOR in Human Osteosarcoma
Source: Int J Mol Sci. 2015 Dec 2;16(12):28635–46. doi: 10.3390/ijms161226123 (PMC4691070; doi:10.3390/ijms161226123)
Supplement: Supplementary file 1 [file ijms-16-26123-s001.pdf]

Supplementary Materials: Overexpression of X-Box Binding Protein 1 (XBP1) Correlates to Poor Prognosis and Up-Regulation of PI3K/mTOR in Human Osteosarcoma

Jielai Yang, Dongdong Cheng, Shumin Zhou, Bin Zhu, Tu Hu and Qingcheng Yang

Table S1. The sequences of used primers.

Gene	Forward Primer	Reverse Primer
XBP1u	5′-AGGAGTTAACACAGCGCTTGGGGATGGAT-3′	5′-CTGAATCTGAAGAGTCAATACCGCCAGAAT-3′
XBP1s	5′-CCTGGTTGCTGAAGAGGAGG-3′	5′-CCATGGGGAGATGTTCTGGAG-3′
HIF-1α	5′-CCGAATTGATGGGATATGAG-3′	5′-TCATGATGAGTTTTGGTCAGATG-3′
PIK3R3	5′-CTTGCTCTGTGGTGGCCGAT-3′	5′-GACGTTGAGGGAGTCGTTGT-3′
mTOR	5′-ATGCAGCTGTCCTGGTTCTC-3′	5′-AATCAGACAGGCACGAAGGG-3′
β-Actin	5′-CTCCATCCTGGCCTCGCTGT-3′	5′-GCTGTCACCTTCACCGTTCC-3′

XBP1u: un-spliced XBP1; XBP1s: spliced XBP1.

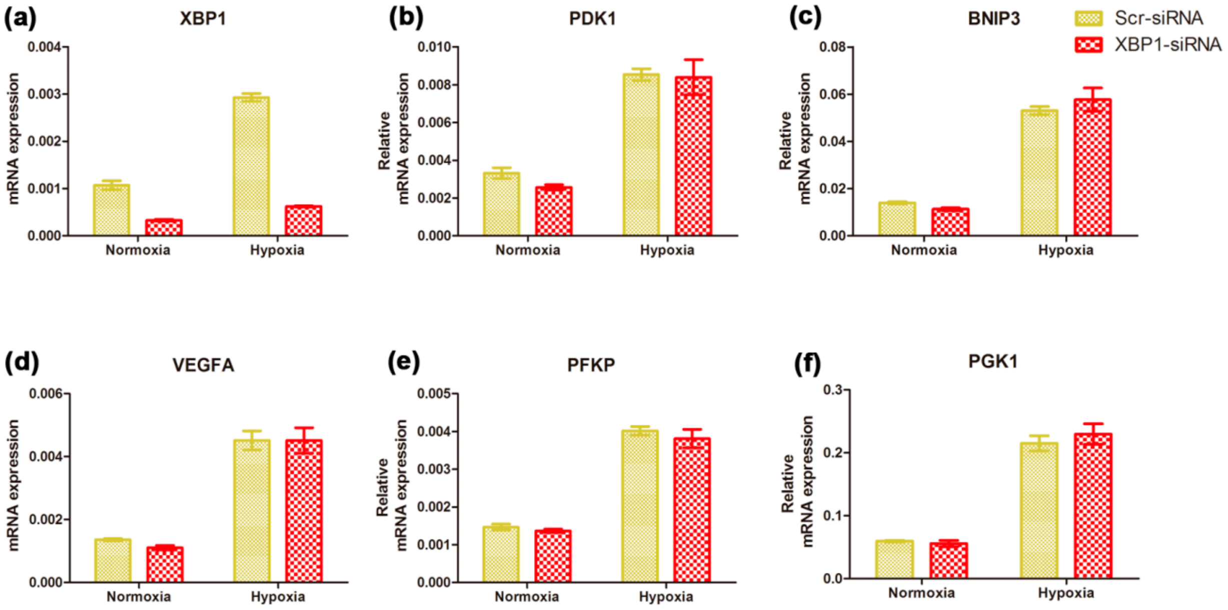

Figure S1. Knockdown of XBP1 did not change the expression of HIF-1-targeted genes. Cells were transfected with either scramble or XBP1 siRNA and exposed to either 21% or 1% oxygen for 24 h. (**a**) Confirmation of XBP1 knockdown by qRT-PCR; (**b**–**f**) Analyses of the expression levels of HIF-1-targeted genes PDK1 (**b**), BNIP3 (**c**), VEGFA (**d**), PFKP (**e**), and PGK1 (**f**) in U2OS cells by qRT-PCR.
